# Supplementary figures and images for: Reservoir host immunology and life history shape virulence evolution in zoonotic viruses
Source: PLoS Biol. 2023 Sep 7;21(9):e3002268. doi: 10.1371/journal.pbio.3002268 (PMC10484437; doi:10.1371/journal.pbio.3002268)

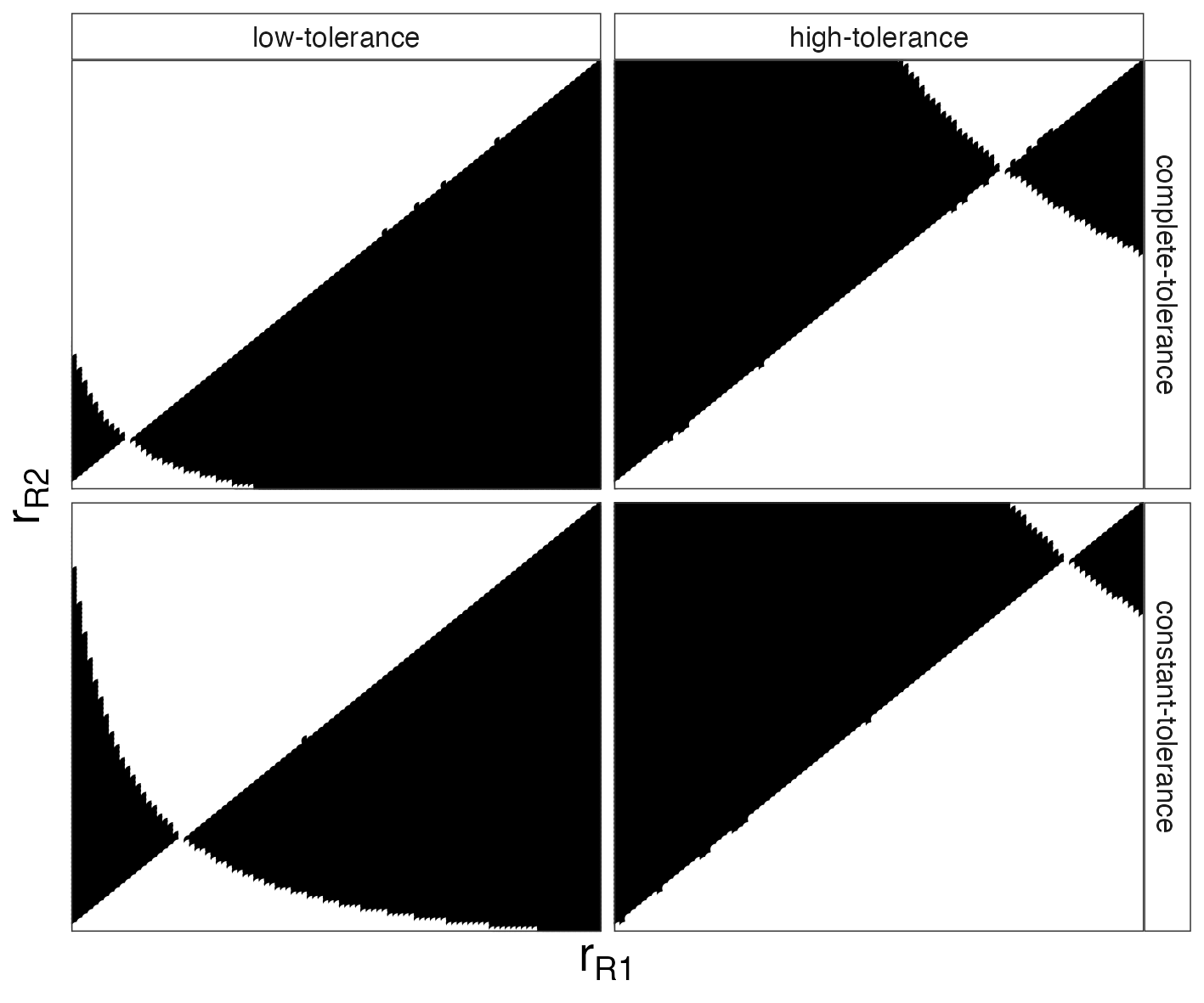

Supplement: S1 Fig — Invading growth rates (rR2) will displace resident growth rates (rR1) at values indicated by the shaded regions. Reservoir host tolerance of immunopathology (TwR) and tolerance of direct virus pathology (TvR) are both modeled as low in left column (0.5 and 10 for row 1 and 2, respectively) and high in right column (0.97 and 100 for row 1 and 2), assuming a complete (row 1) or a constant form (row 2). For this visualization, rR1 and rR2 span from 3.18 to 3.5. All other parameters involved in computation of rR* (see S1 File equations [25,26]) were fixed at values listed in Table 1 (main text). Data and code used to generate all figure panels are available in our publicly available GitHub repository (github.com/brooklabteam/spillover-virulence-v1.0.0; doi: 10.5281/zenodo.8136864). (PNG) [file pbio.3002268.s001.png]

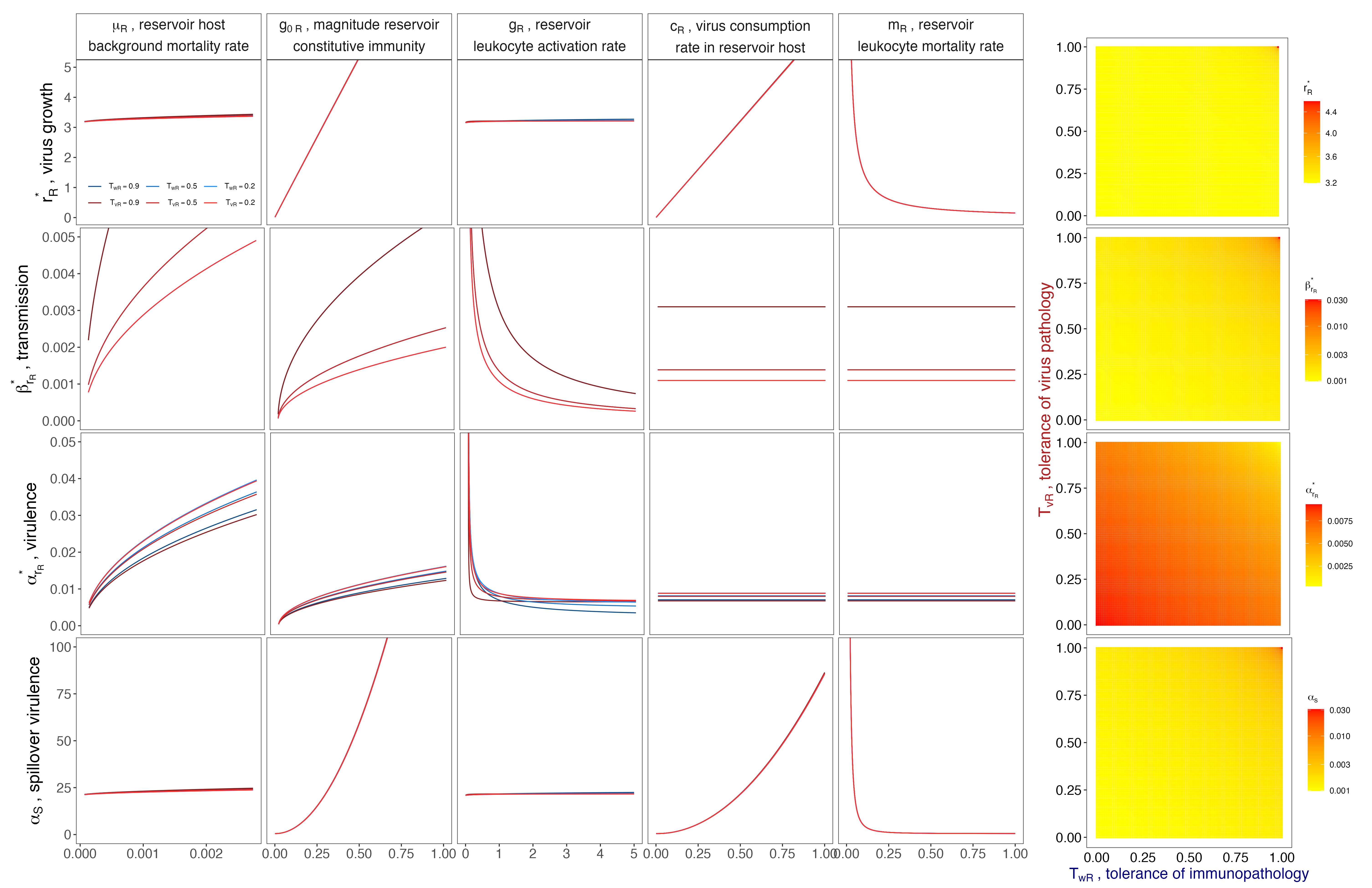

Supplement: S2 Fig — Figure replicates Fig 2 (main text) under assumptions of complete tolerance. Rows (top-down) indicate the evolutionarily optimal within-host virus growth rate (rR*) and the corresponding transmission rate (βrR*), and virus-induced mortality rate (αrR*) for a reservoir host infected with a virus at rR*. The bottom row then demonstrates the resulting virulence (αS) of a reservoir-optimized virus evolved to rR* upon nascent spillover to a novel, secondary host. Columns demonstrate the dependency of these outcomes on variable reservoir host parameters: background mortality rate (μR), extent of constitutive immunity (g0R), leukocyte activation rate upon viral contact (gR), virus consumption rate by leukocytes (cR), leukocyte mortality rate (mR). Darker colored lines depict outcomes at higher values for reservoir host tolerance of virus pathology (TvR, red) or immunopathology (TwR, blue), assuming no tolerance of the opposing type. Heat maps demonstrate how TvR and TwR interact to produce each outcome. Outcome ranges differ between lineplots (y-axes) and heat maps (scale bars). Parameter values are listed in Table 1 (main text). Data and code used to generate all figure panels are available in our publicly available GitHub repository (github.com/brooklabteam/spillover-virulence-v1.0.0; doi: 10.5281/zenodo.8136864). (PNG) [file pbio.3002268.s002.png]

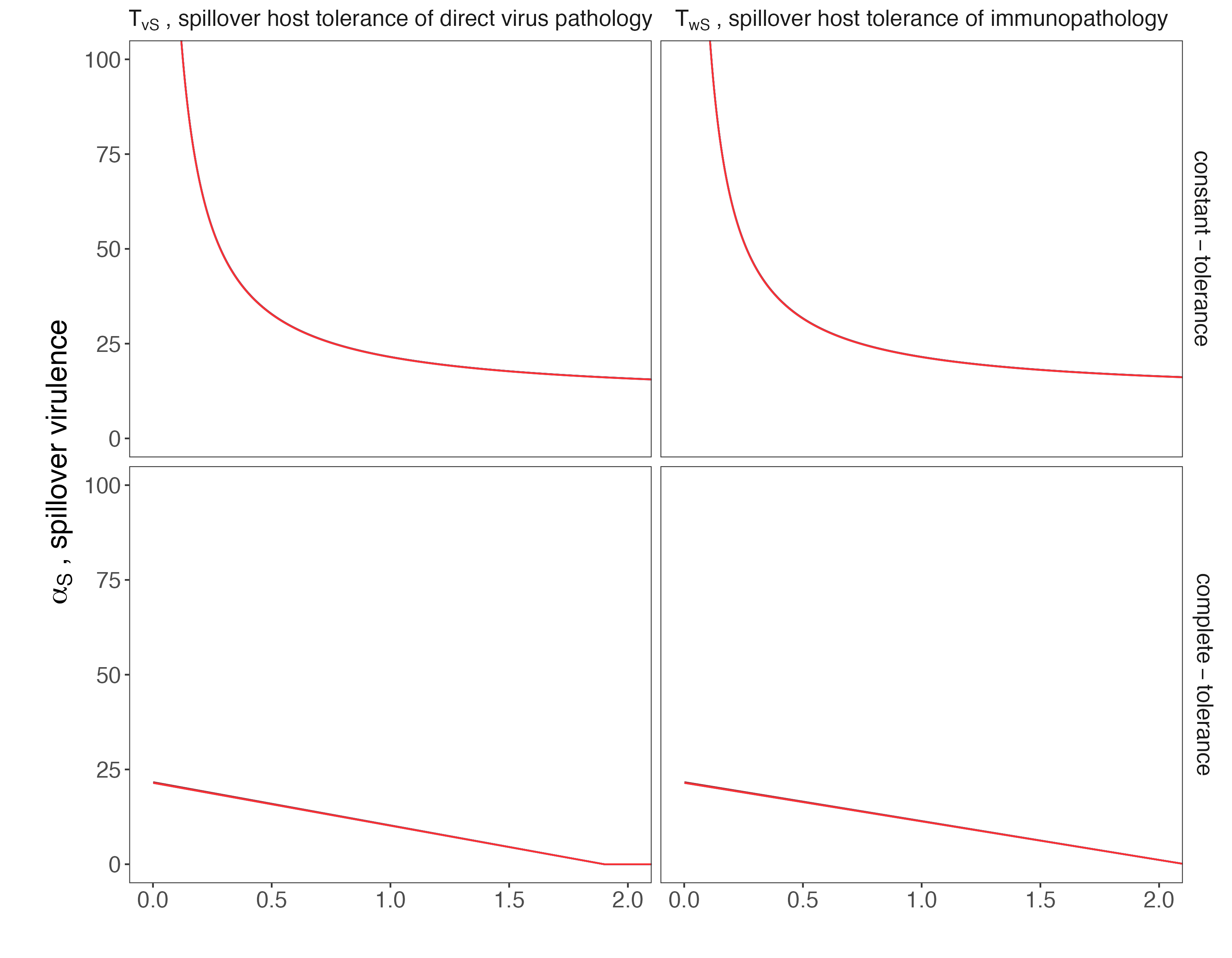

Supplement: S3 Fig — Virulence of spilled-over virus (αS) across a range of values for 2 mechanisms of tolerance in the spillover host: tolerance of direct virus pathology (TvS, left column) and tolerance of immunopathology (TwS, right column). Results are expressed under assumptions of constant tolerance (top panels) and complete tolerance (bottom panels). In main text results, TwS is held constant for all predictions of spillover virulence but TvS is varied proportionally to the inverse time to MRCA between reservoir and spillover host. Data and code used to generate all figure panels are available in our publicly available GitHub repository (github.com/brooklabteam/spillover-virulence-v1.0.0; doi: 10.5281/zenodo.8136864). (PNG) [file pbio.3002268.s003.png]

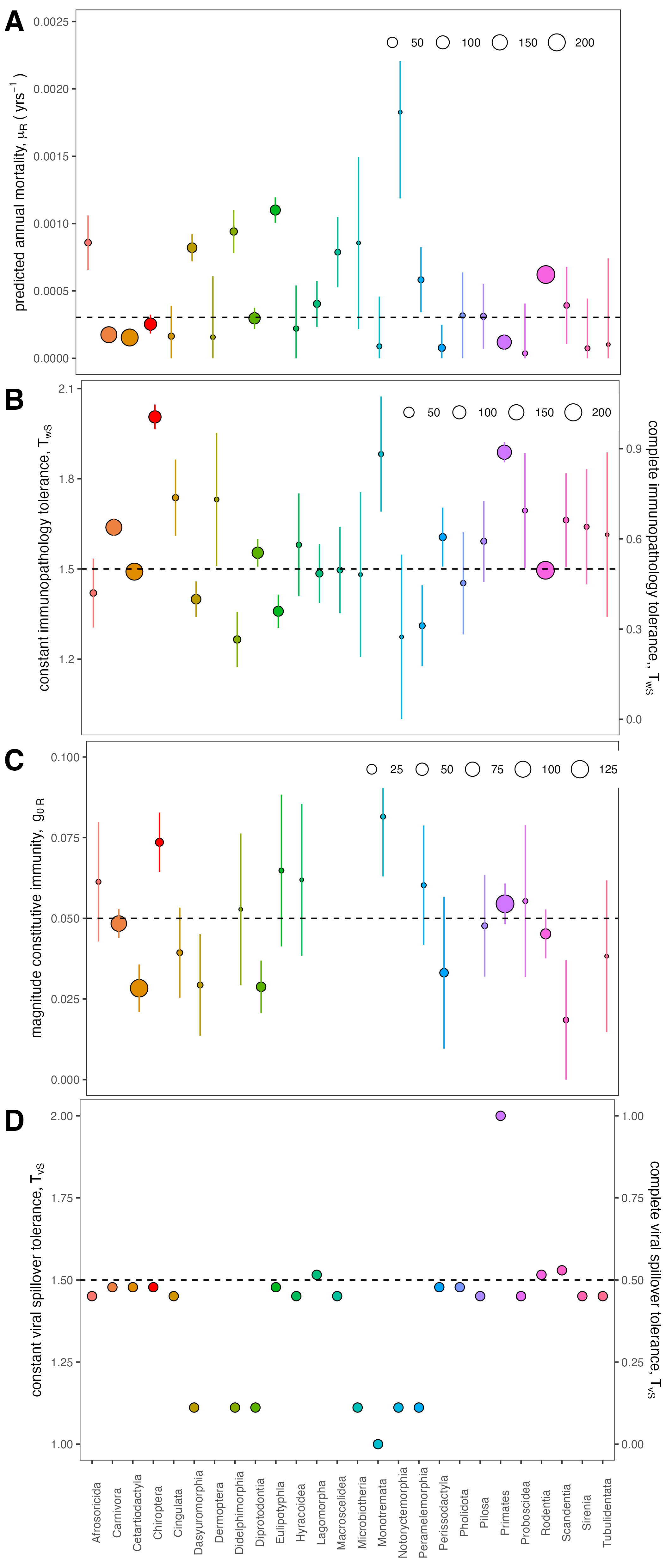

Supplement: S4 Fig — Model parameter estimates for (A) reservoir-host background mortality (μR), (B) tolerance of immunopathology (TwR) (left y-axis: constant tolerance assumptions; right y-axis: complete tolerance assumptions), (C) magnitude of constitutive immunity (g0R), and (D) magnitude of human tolerance of virus pathology for a virus evolved in a disparate mammalian reservoir (TvS). Estimates are derived from (A) linear model predictions of maximum lifespan at the order level, (B) the scaled effect of order on a linear mixed model prediction of lifespan per body size, (C) the scaled effect of order on a linear mixed model prediction of neutrophil concentration for mass-specific BMR, and (D) the magnitude of human tolerance of virus pathology for a virus evolved in a disparate mammalian reservoir (TvS), corresponding to data presented in Fig 3E (main text). Default parameter values involved in the estimation process are summarized in Table 1 (main text), and estimated parameters and corresponding 95% confidence intervals by standard error are presented in S1 Table. See main text Methods and our open-source GitHub repository for a detailed walk-through of the parameter estimation process. Data and code used to generate all figure panels are available in our publicly available GitHub repository (github.com/brooklabteam/spillover-virulence-v1.0.0; doi: 10.5281/zenodo.8136864). (PNG) [file pbio.3002268.s004.png]

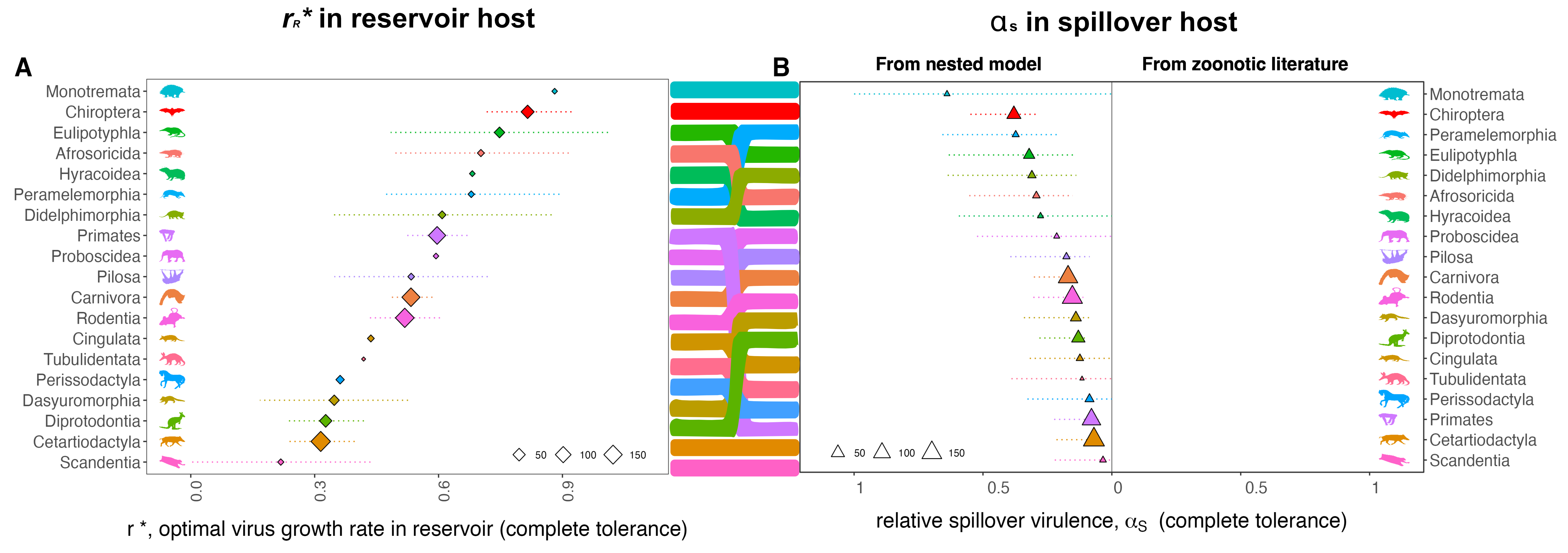

Supplement: S5 Fig — Figure replicates Fig 3D and 3G from the main text, here under assumptions of complete tolerance. Panel (A) depicts optimal rR* across 19 mammalian orders, for which we were able to estimate order-level specific values for the 3 within-host reservoir parameters which we varied in our analysis (μR, TwR, and g0R; visualized in S4 Fig), while panel (B) depicts the resulting estimation of relative spillover virulence (αS), which also relies on order-specific values for the spillover host tolerance of direct virus pathology (TvS). Taxa in panels (A) and (B) are arranged in descending order from highest to lowest predicted values for, respectively rR* and αS. This order varies slightly from panel (A) to (B), as highlighted by alluvial flows and discussed in the main text. See S1 Table for order-level values for rR*, μR, TwR, g0R, and TvS and Table 1 (main text) for all other parameters involved in calculation of rR* and αS. Data and code used to generate all figure panels are available in our publicly available GitHub repository (github.com/brooklabteam/spillover-virulence-v1.0.0; doi: 10.5281/zenodo.8136864). (PNG) [file pbio.3002268.s005.png]

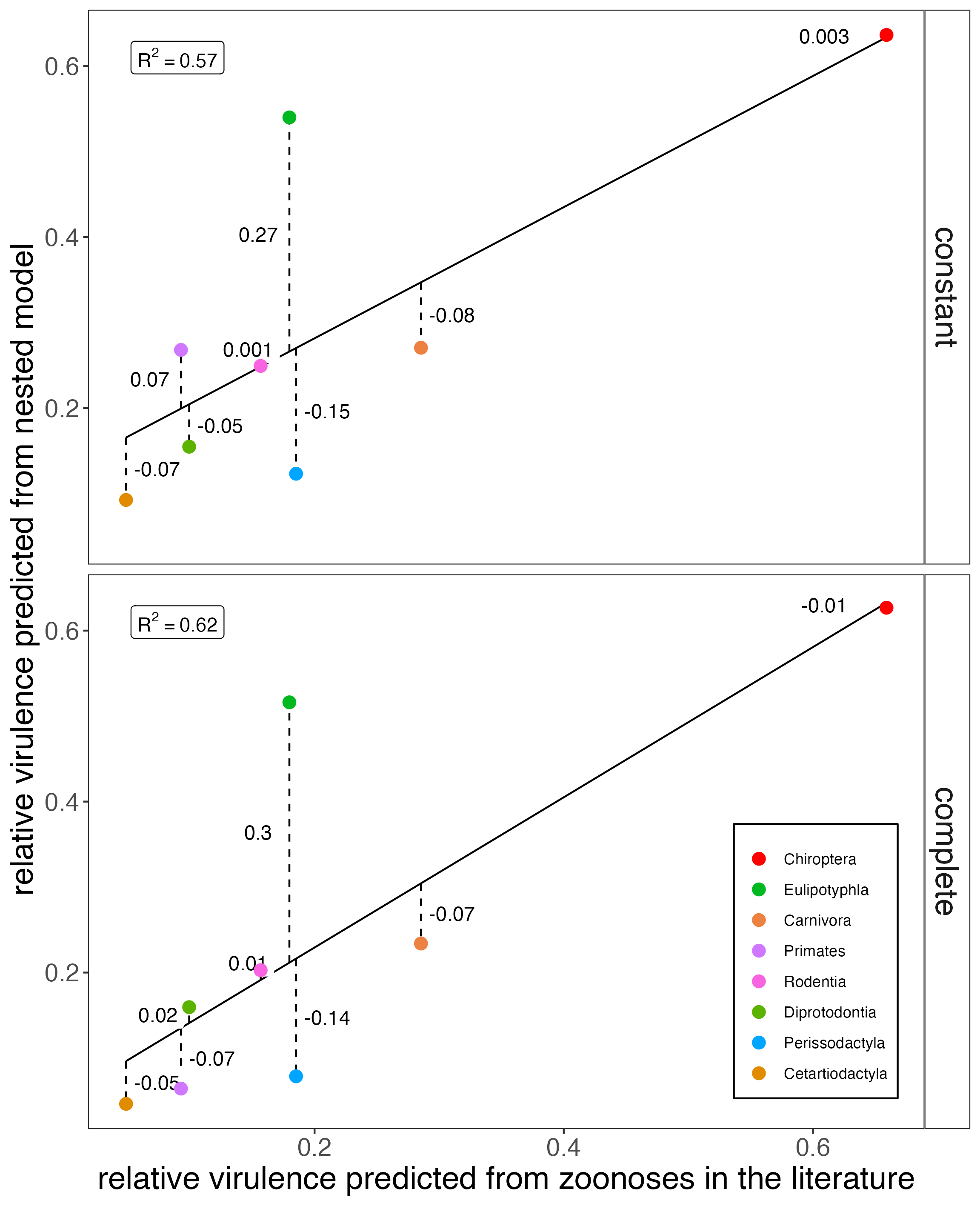

Supplement: S6 Fig — Figure plots observed vs. predicted spillover virulence for 8 orders from Fig 3G (main text) for which case fatality rates from corresponding zoonoses are reported in the literature [8]. Panel (A) compares nested modeling predictions under assumptions of constant tolerance with those from the literature, while panel (B) does the same under assumptions for complete tolerance. In both cases, a fitted linear regression and corresponding R2 value is shown as a quantitative evaluation of model fit to the data. Dashed lines give the residual of each data point from the regression line. Data and code used to generate all figure panels are available in our publicly available GitHub repository (github.com/brooklabteam/spillover-virulence-v1.0.0; doi: 10.5281/zenodo.8136864). (PNG) [file pbio.3002268.s006.png]

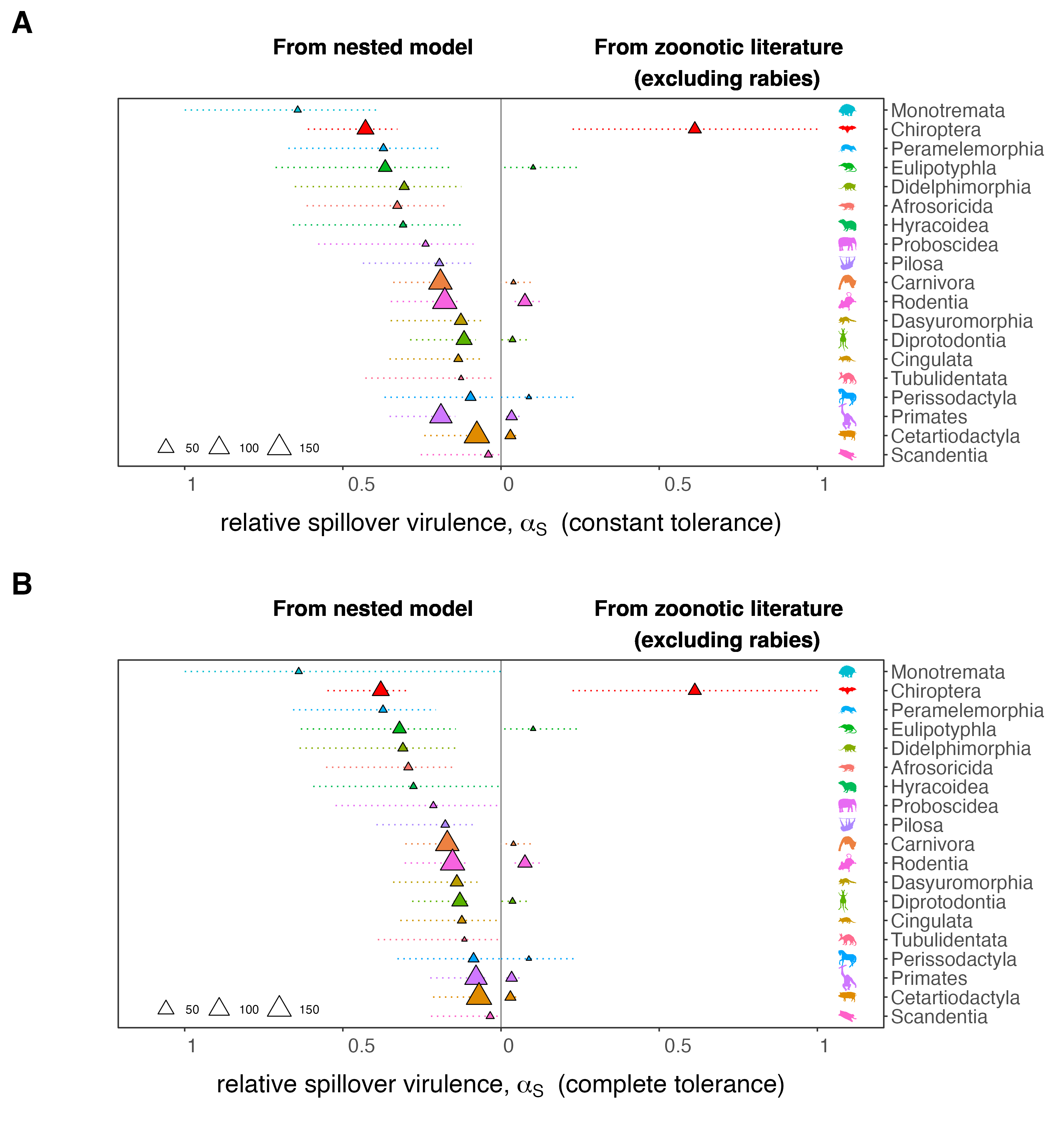

Supplement: S7 Fig — Figure replicates Fig 3G (main text), respectively, under assumptions of (A) constant and (B) complete tolerance but excluding rabies lyssavirus from the zoonotic data (right-half of panels). Rank-order predictions of virulence are more consistent with order Carnivora further down in the rankings. As in Fig 3G, order-specific parameter values for rR*, μR, TwR, g0R, and TvS are listed in S1 Table; all other parameters involved in calculation of αS are listed in Table 1 (main text). Data and code used to generate all figure panels are available in our publicly available GitHub repository (github.com/brooklabteam/spillover-virulence-v1.0.0; doi: 10.5281/zenodo.8136864). (PNG) [file pbio.3002268.s007.png]

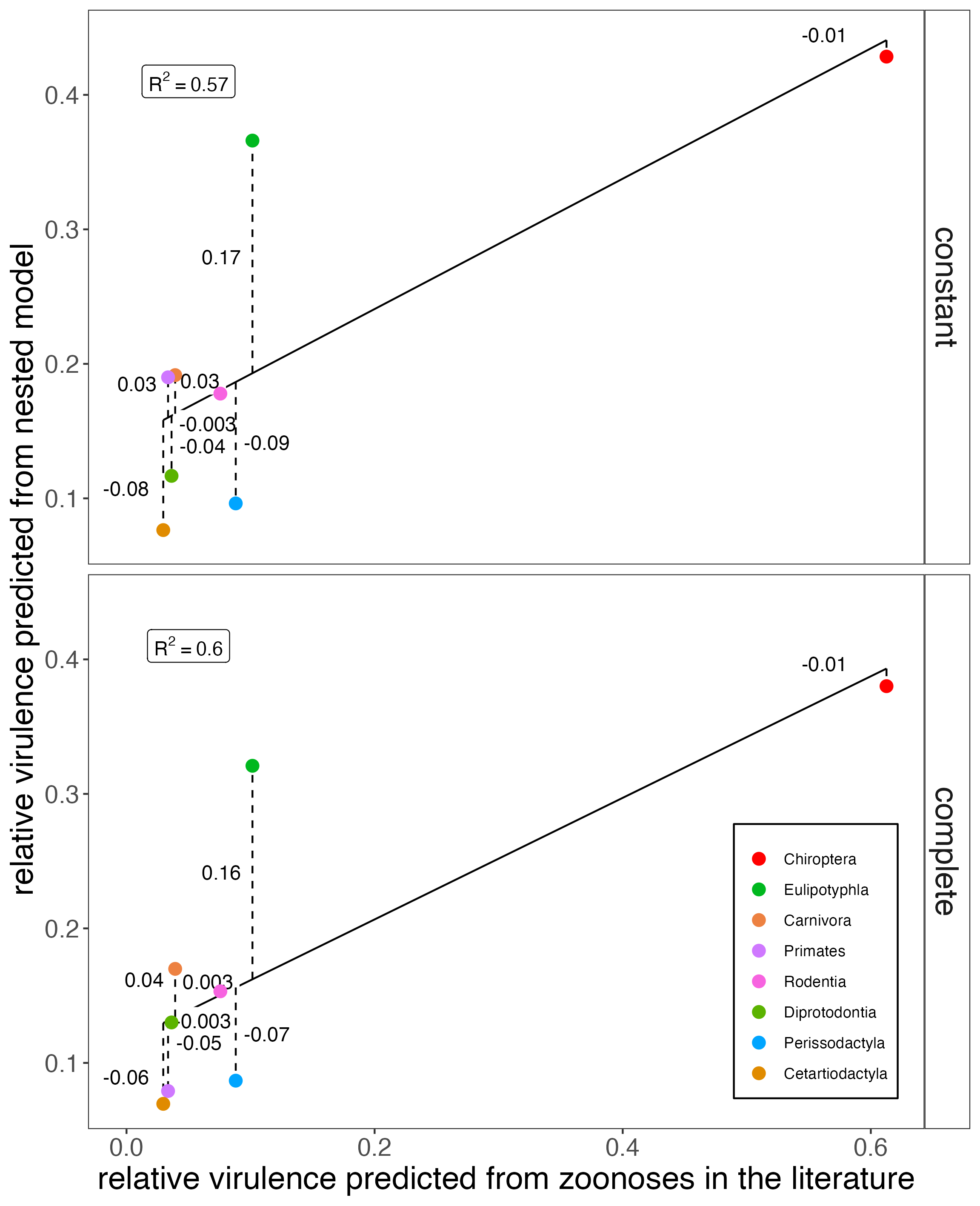

Supplement: S8 Fig — Plot recapitulates S6 Fig exactly, but comparisons are drawn from case fatality rates reported in the literature but excluding rabies lyssavirus, which is often classed as a Carnivora-derived virus, though its evolutionary origins are found in bats. Removal of rabies improves estimates of virulence for Carnivora-derived zoonoses as compared with the complete dataset, but resulting linear regression offers no better fit to the entire dataset than previously shown in S6 Fig. Data and code used to generate all figure panels are available in our publicly available GitHub repository (github.com/brooklabteam/spillover-virulence-v1.0.0; doi: 10.5281/zenodo.8136864). (PNG) [file pbio.3002268.s008.png]

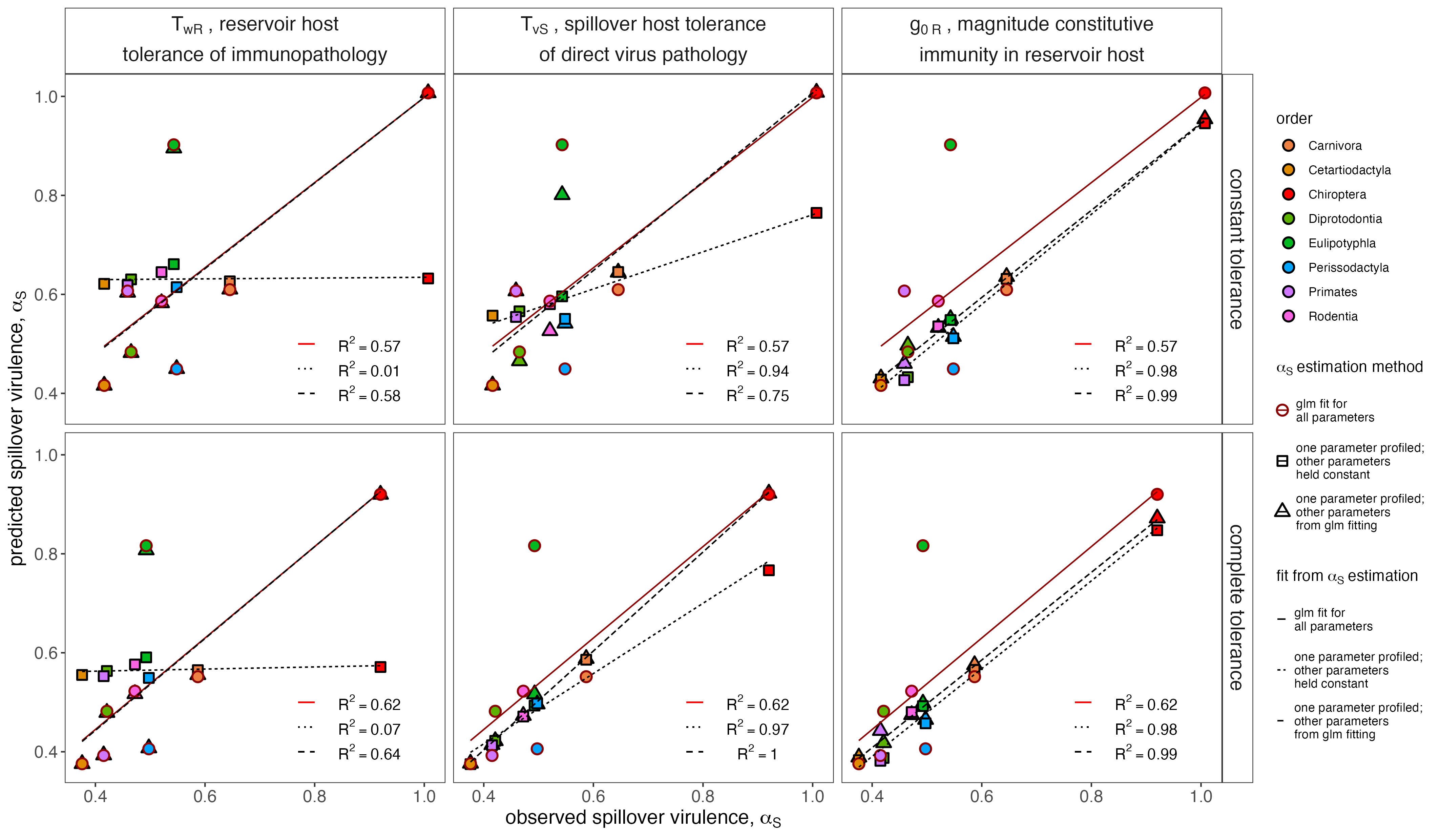

Supplement: S9 Fig — Figure replicates S6 Fig in part with observed spillover virulence (αS) from case fatality rates reported in the literature [8] depicted on the x-axis and predictions from nested modeling framework on the y-axis. In all panels, circles correspond to nested modeling predictions of spillover virulence using parameter values recovered from regression analysis of publicly available life history data as presented in the main text, replicating points from S6 Fig. Projections from nested modeling approach assuming constant tolerance are shown in the top panels and complete tolerance in the bottom. In lieu of life history-derived parameter values, squares show αS estimates from nested model using constant, universal values across all orders for all parameters excepting the parameter profiled in the corresponding column (TwR, TvS, or g0R). When not profiled, TwR = 1.5 (constant) and 0.5 (complete); TvS = 1.5 (constant) and 0.5 (complete); and g0R = 0.5 for simulations resulting in square points. Finally, triangles give αS estimates from nested model approach using parameters generated by profiling the parameter in the corresponding column (TwR, TvS, or g0R), while pairing it with values recovered using regression analysis the literature for other variable parameters (S1 Table). Lines and corresponding R2 values signify the fit of a simple linear regression of observed vs. predicted αS across all mammalian orders, where predicted values are generated from nested modeling approach using: linear regression analysis of life history data for TwR, TvS, and g0R (solid line, red, same as reported in the main text); profiling TwR, TvS, or g0R while holding constant all other parameters across orders (thin dashed line); and profiling TwR, TvS, or g0R while using linear regression estimates from life history data for parameters not being profiled (thick dashed line). Data and code used to generate all figure panels are available in our publicly available GitHub repository (github.com/b [file pbio.3002268.s009.png]
