# Supplementary material for: Reservoir host immunology and life history shape virulence evolution in zoonotic viruses
Source: PLoS Biol. 2023 Sep 7;21(9):e3002268. doi: 10.1371/journal.pbio.3002268 (PMC10484437; doi:10.1371/journal.pbio.3002268)
Supplement: S1 Table — (PDF) [file pbio.3002268.s010.pdf]

Table S1. Order-specific parameter values for within-host nested model.*

	μ_R, reservoir host background mortality (days^{-1})	T_{wR}, reservoir host tolerance of immunopathology *(constant)*	T_{wR}, reservoir host tolerance of mmunopathology *(complete)*	g_{0R}, reservoir host magnitude of constitutive immunity (days^{-1})	T_{vS}, human tolerance of viral pathology *(constant)*	T_{vS}, human tolerance of viral pathology *(complete)*	r_R^*, optimal virus growth rate (days^{-1}) *(constant)*	r_R^*, optimal virus growth rate (days^{-1}) *(complete)*	α_S, relative spillover virulence (days^{-1}) *(constant)*	α_S, relative spillover virulence (days^{-1}) *(complete)*
Afrosoricida	$8.58x10^{4}$ [$6.56x10^{4}$-$1.06x10^{3}$]	1.42 [1.31-1.53]	0.42 [0.305-0.535]	0.0613 [0.0428-0.0798]	1.45	0.451	0.7 [0.49-0.911]	0.703 [0.491-0.915]	0.328 [0.168-0.615]	0.293 [0.152-0.553]
Carnivora	$1.75x10^{4}$ [$1.26x10^{4}$-$2.24x10^{4}$]	1.64 [1.61-1.67]	0.638 [0.609-0.667]	0.0484 [0.0439-0.0529]	1.48	0.478	0.531 [0.48-0.583]	0.533 [0.481-0.585]	0.192 [0.159-0.341]	0.17 [0.141-0.303]
Cetartiodactyla	$1.54x10^{4}$ [$1.09x10^{4}$-$2.00x10^{4}$]	1.49 [1.46-1.52]	0.491 [0.464-0.518]	0.0283 [0.021-0.0357]	1.48	0.478	0.314 [0.232-0.396]	0.315 [0.233-0.397]	0.0764 [0.0478-0.243]	0.0696 [0.0447-0.215]
Chiroptera	$2.54x10^{4}$ [$1.83x10^{4}$-$3.24x10^{4}$]	2.01 [1.96-2.05]	1.01 [0.964-1.05]	0.0736 [0.0644-0.0828]	1.48	0.478	0.808 [0.704-0.912]	0.816 [0.71-0.922]	0.428 [0.327-0.611]	0.38 [0.29-0.549]
Cingulata	$1.64x10^{4}$ [0-$3.90x10^{4}$]	1.74 [1.61-1.86]	0.737 [0.61-0.864]	0.0394 [0.0254-0.0533]	1.45	0.451	0.434 [0.267-0.597]	0.436 [0-0.603]	0.135 [0.0609-0.353]	0.124 [0-0.319]
Dasyuromorphia	$8.21x10^{4}$ [$7.20x10^{4}$-$9.22x10^{4}$]	1.4 [1.34-1.46]	0.399 [0.34-0.458]	0.0293 [0.0136-0.0451]	1.11	0.111	0.346 [0.167-0.524]	0.347 [0.167-0.526]	0.127 [0.0587-0.349]	0.14 [0.0744-0.341]
Dermoptera	$1.57x10^{4}$ [0-$6.09x10^{4}$]	1.73 [1.51-1.95]	0.731 [0.509-0.953]	---	---	---	---	---	---	---
Didelphimorphia	$9.41x10^{4}$ [$7.81x10^{4}$-$1.10x10^{3}$]	1.27 [1.17-1.36]	0.265 [0.173-0.357]	0.0528 [0.0293-0.0763]	1.11	0.111	0.608 [0.343-0.872]	0.609 [0.343-0.874]	0.306 [0.125-0.652]	0.31 [0.138-0.637]
Diprotodontia	$2.97x10^{4}$ [$2.18x10^{4}$-$3.75x10^{4}$]	1.55 [1.51-1.6]	0.554 [0.507-0.6]	0.0288 [0.0206-0.0369]	1.11	0.111	0.325 [0.233-0.417]	0.327 [0.234-0.419]	0.117 [0.0794-0.288]	0.13 [0.0942-0.281]
Eulipotyphla	$1.10x10^{3}$ [$1.01x10^{3}$-$1.19x10^{3}$]	1.36 [1.3-1.41]	0.359 [0.304-0.415]	0.0648 [0.0413-0.0883]	1.48	0.478	0.746 [0.483-1.01]	0.748 [0.484-1.01]	0.366 [0.161-0.713]	0.321 [0.143-0.633]
Hyracoidea	$2.21x10^{4}$ [0-$5.41x10^{4}$]	1.58 [1.41-1.75]	0.58 [0.409-0.751]	0.0619 [0.0384-0.0855]	1.45	0.451	0.68 [0.403-0.952]	0.682 [0-0.959]	0.31 [0.119-0.658]	0.277 [0-0.594]
Lagomorpha	$4.04x10^{4}$ [$2.33x10^{4}$-$5.75x10^{4}$]	1.48 [1.39-1.58]	0.485 [0.387-0.583]	---	1.52	0.516	---	---	---	---
Macroscelidea	$7.87x10^{4}$ [$5.26x10^{4}$-$1.05x10^{3}$]	1.5 [1.35-1.64]	0.496 [0.352-0.64]	---	1.45	0.451	---	---	---	---
Microbiotheria	$8.56x10^{4}$ [$2.16x10^{4}$-$1.50x10^{3}$]	1.48 [1.21-1.76]	0.481 [0.208-0.755]	---	1.11	0.111	---	---	---	---
Monotremata	$8.88x10^{5}$ [0-$4.58x10^{4}$]	1.88 [1.69-2.07]	0.882 [0.691-1.07]	0.0815 [0.063-0.1]	1	0	0.878 [0.661-1.11]	0.881 [0-1.12]	0.643 [0.384-1]	0.639 [0-1]
Notoryctemorphia	$1.83x10^{3}$ [$1.19x10^{3}$-$2.47x10^{3}$]	1.27 [1-1.55]	0.274 [0-0.548]	---	1.11	0.111	---	---	---	---
Peramelemorphia	$5.83x10^{4}$ [$3.41x10^{4}$-$8.24x10^{4}$]	1.31 [1.18-1.45]	0.311 [0.176-0.446]	0.0603 [0.0417-0.0788]	1.11	0.111	0.678 [0.467-0.89]	0.679 [0.467-0.893]	0.372 [0.197-0.672]	0.373 [0.206-0.658]
Perissodactyla	$7.74x10^{5}$ [0-$2.48x10^{4}$]	1.61 [1.51-1.7]	0.606 [0.508-0.704]	0.0331 [0.00962-0.0567]	1.48	0.478	0.361 [0.101-0.625]	0.362 [0-0.628]	0.0963 [0.0166-0.369]	0.0868 [0-0.328]

Pholidota	3.17x10^{4} [0-6.37x10^{4}]	1.45 [1.28-1.62]	0.453 [0.282-0.624]	---	1.48	0.478	---	---	---	---
Pilosa	3.11x10^{4} [6.93x10^{5}-5.53x10^{4}]	1.59 [1.46-1.73]	0.592 [0.457-0.727]	0.0477 [0.0319-0.0635]	1.45	0.451	0.532 [0.347-0.714]	0.534 [0.348-0.719]	0.195 [0.0923-0.437]	0.176 [0.0857-0.394]
Primates	1.20x10^{4} [6.27x10^{5}-1.77x10^{4}]	1.89 [1.85-1.92]	0.888 [0.855-0.922]	0.0545 [0.0482-0.0608]	2	1	0.593 [0.52-0.665]	0.597 [0.522-0.67]	0.19 [0.144-0.352]	0.0791 [0.0504-0.224]
Proboscidea	3.70x10^{5} [0-4.06x10^{4}]	1.69 [1.5-1.89]	0.694 [0.503-0.886]	0.0553 [0.0318-0.0789]	1.45	0.451	0.593 [0.334-0.875]	0.594 [0-0.882]	0.238 [0.0868-0.578]	0.214 [0-0.523]
Rodentia	6.21x10^{4} [5.79x10^{4}-6.64x10^{4}]	1.5 [1.47-1.52]	0.496 [0.47-0.521]	0.0452 [0.0376-0.0528]	1.52	0.516	0.516 [0.431-0.601]	0.518 [0.433-0.603]	0.178 [0.128-0.349]	0.153 [0.11-0.305]
Scandentia	3.92x10^{4} [1.06x10^{4}-6.78x10^{4}]	1.66 [1.51-1.82]	0.662 [0.507-0.818]	0.0185 [1x107-0.037]	1.53	0.529	0.216 [2.68x105-0.43]	0.218 [2.81x105-0.435	0.04 [0-0.254]	0.0343 [0-0.222]
Sirenia	7.39x10^{5} [0-4.43x10^{4}]	1.64 [1.45-1.83]	0.64 [0.449-0.832]	---	1.45	0.451	---	---	---	---
Tubulidentata	1.02x10^{4} [0-7.42x10^{4}]	1.61 [1.34-1.89]	0.614 [0.34-0.888]	0.0382 [0.0147-0.0618]	1.45	0.451	0.417 [0.155-0.704]	0.419 [0-0.713]	0.126 [0.029-0.429]	0.116 [0-0.39]

*Derived parameter values (depicted as *mean [lower confidence interval – upper confidence interval]*) computed from linear regression and mixed effects models are shown across all 26 mammalian orders ($\mu_R, g_{0R}, T_{wR}, T_{vS}$), in addition to the r_R^* and α_S values computed from their inputs. Confidence intervals are 95% derived via standard error. All other parameters involved in the calculation of r_R^* and α_S are listed in main text Table 1.
