# Supplementary material for: Reservoir host immunology and life history shape virulence evolution in zoonotic viruses
Source: PLoS Biol. 2023 Sep 7;21(9):e3002268. doi: 10.1371/journal.pbio.3002268 (PMC10484437; doi:10.1371/journal.pbio.3002268)
Supplement: S2 Table — (PDF) [file pbio.3002268.s011.pdf]

S2 Table. Model summary outputs for within-host parameter estimation.*

A. Summary of linear regression model to predict $\boldsymbol{\mu_R}$

Formula: *lm(mortality_rate~order, data = pan.dat);* **Number observations:** 1060; $\mathbf{R^2}$=0.42

Term	Estimate	Lci-Uci	Statistic	P-value
Intercept (Diprotodontia)	0.11	0.08 - 0.14	T: 7.38	<0.001***
Rodentia	0.12	0.09 - 0.15	T: 7.1	<0.001***
Carnivora	-0.04	0-0.07	T: -2.58	0.01**
Cetartiodactyla	-0.05	0-0.07	T: -3.07	<0.001***
Primates	-0.06	0-0.07	T: -3.56	<0.001***
Afrosoricida	0.2	0.13 - 0.28	T: 5.07	<0.001***
Chiroptera	-0.02	0-0.07	T: -0.8	0.42
Dasyuromorphia	0.19	0.14 - 0.24	T: 8.02	<0.001***
Eulipotyphla	0.29	0.25 - 0.34	T: 12.82	<0.001***
Pilosa	0.01	0-0.19	T: 0.11	0.91
Didelphimorphia	0.24	0.17 - 0.3	T: 7.09	<0.001***
Cingulata	-0.05	0-0.18	T: -1.09	0.28
Perissodactyla	-0.08	0-0.14	T: -2.28	0.02*
Dermoptera	-0.05	0-0.34	T: -0.6	0.55
Hyracoidea	-0.03	0-0.24	T: -0.45	0.65
Microbiotheria	0.2	0-0.47	T: 1.7	0.09
Sirenia	-0.08	0-0.28	T: -1.16	0.25
Peramelemorphia	0.1	0.01 - 0.2	T: 2.2	0.03
Macroscelidea	0.18	0.08 - 0.28	T: 3.53	<0.001***
Proboscidea	-0.09	0-0.27	T: -1.35	0.18
Lagomorpha	0.04	0-0.14	T: 1.12	0.26
Pholidota	0.01	0-0.24	T: 0.12	0.9
Notoryctemorphia	0.56	0.32 - 0.79	T: 4.65	<0.001***
Monotremata	-0.08	0-0.27	T: -1.08	0.28
Tubulidentata	-0.07	0-0.47	T: -0.59	0.55
Scandentia	0.03	0-0.21	T: 0.63	0.53

B. Summary of linear mixed effect regression model to estimate $\boldsymbol{T_{wR}}$

Formula: *lmer(log10_max_lifespan_yrs ~ log10mass_g + (1 | order), data = pan.dat)*
Number observations: 1060; **Groups:** 26

Term	Estimate	Lci-Uci	Statistic	P-value
(Intercept)	0.392	0.295-0.488	T: 7.96	<0.001***
log10mass (grams)	.199	0.185-0.214	T: 26.55	<0.001***
order (random effect)	Variance: 0.041	Std Dev: 0.202		

C. Summary of linear mixed effect regression model to estimate $\boldsymbol{g_0}$

Formula: *lmer(log10_neutrophil_count ~ log10mass_g + BMR_W + (1 | order), data = pan.dat)*
Number observations: 144; **Groups**: 19

Term	Estimate	Lci-Uci	Statistic	P-value
(Intercept)	-0.245	-0.422 - -068	T: 2.72	0.008**
log10mass (grams)	0.212	0.168-0.255	T: 9.53	<0.001***
BMR (W)	-0.000368	-0.000103- - 0.000633	T: -2.72	0.01*
order (random effect)	Variance: 0.041	Std Dev: 0.203		

D. Summary of generalized additive model to summarize $\boldsymbol{\alpha_S}$ by order from literature

Formula: *alpha ~ s(vFamily, bs = "re") + s(hOrder, bs = "re") + s(VirusPubs, k = 7, bs = "tp") + s(spill_type, bs = "re") + s(IsVectorBorne, bs = "re");* **Deviance explained:** 88.2%; $\mathbf{R^2}$**:** 0.873; **N** = 75

Term	Estimate	Lci-Uci	Statistic	P-value	Effective degrees of freedom
Intercept<br>*smoothing:*	-4.55	[-6.15 – -2.95]	*z:* -5.57	<.001***	
virus family			χ^2: 612	.00387**	9.50
order			χ^2: 1161	<.001***	4.74
virus spp. pub count			χ^2: 5.77	.0163*	1.00
spillover type			χ^2: 67.9	.00856**	0.867
vector-borne status			χ^2: 98.9	.0249*	0.806

*Outputs from fitted linear and linear mixed effect regression models used to generate within-host parameter values. Projections for model A and order-level effects in B and C are visualized in main text Fig 3C. Order-level predictions for each parameter are visualized in S4 Fig and presented in S1 Table. Significance at p < 0.001***, 0.01**, 0.05*.
