# Supplementary material for: Reservoir host immunology and life history shape virulence evolution in zoonotic viruses
Source: PLoS Biol. 2023 Sep 7;21(9):e3002268. doi: 10.1371/journal.pbio.3002268 (PMC10484437; doi:10.1371/journal.pbio.3002268)
Supplement: S3 Table — (PDF) [file pbio.3002268.s012.pdf]

S3 Table. Sensitivity analysis and comparison of influence of individual parameter estimates on overall fit of nested model to data.

Order	Tolerance	Fitted Par	Fit Type*	Par Value (this fit)	Par Value (glm fit)	α_S (this fit)	α_S (glm fit)	α_S (lit)
Carnivora	constant	T_{wR}	one profiled; rest of parameters constant	2	1.64	0.63	0.61	0.65
Carnivora	constant	T_{vS}	one profiled; rest of parameters constant	1.39	1.48	0.65	0.61	0.65
Carnivora	constant	g_{0R}	one profiled; rest of parameters constant	0.05	0.05	0.63	0.61	0.65
Carnivora	constant	T_{wR}	one profiled; glm fit rest of parameters	2	1.64	0.61	0.61	0.65
Carnivora	constant	T_{vS}	one profiled; glm fit rest of parameters	1.3	1.48	0.64	0.61	0.65
Carnivora	constant	g_{0R}	one profiled; glm fit rest of parameters	0.05	0.05	0.64	0.61	0.65
Cetartiodactyla	constant	T_{wR}	one profiled; rest of parameters constant	1	1.49	0.62	0.42	0.42
Cetartiodactyla	constant	T_{vS}	one profiled; rest of parameters constant	2	1.48	0.56	0.42	0.42
Cetartiodactyla	constant	g_{0R}	one profiled; rest of parameters constant	0.03	0.03	0.43	0.42	0.42
Cetartiodactyla	constant	T_{wR}	one profiled; glm fit rest of parameters	1.49	1.49	0.42	0.42	0.42
Cetartiodactyla	constant	T_{vS}	one profiled; glm fit rest of parameters	1.47	1.48	0.42	0.42	0.42
Cetartiodactyla	constant	g_{0R}	one profiled; glm fit rest of parameters	0.03	0.03	0.43	0.42	0.42
Chiroptera	constant	T_{wR}	one profiled; rest of parameters constant	2	2.01	0.63	1.01	1.01
Chiroptera	constant	T_{vS}	one profiled; rest of parameters constant	1	1.48	0.76	1.01	1.01
Chiroptera	constant	g_{0R}	one profiled; rest of parameters constant	0.07	0.07	0.95	1.01	1.01
Chiroptera	constant	T_{wR}	one profiled; glm fit rest of parameters	2	2.01	1.01	1.01	1.01
Chiroptera	constant	T_{vS}	one profiled; glm fit rest of parameters	1.47	1.48	1.01	1.01	1.01
Chiroptera	constant	g_{0R}	one profiled; glm fit rest of parameters	0.07	0.07	0.95	1.01	1.01
Diprotodontia	constant	T_{wR}	one profiled; rest of parameters constant	1	1.55	0.63	0.48	0.47
Diprotodontia	constant	T_{vS}	one profiled; rest of parameters constant	2	1.11	0.57	0.48	0.47
Diprotodontia	constant	g_{0R}	one profiled; rest of parameters constant	0.03	0.03	0.43	0.48	0.47
Diprotodontia	constant	T_{wR}	one profiled; glm fit rest of parameters	1	1.55	0.48	0.48	0.47
Diprotodontia	constant	T_{vS}	one profiled; glm fit rest of parameters	1.2	1.11	0.47	0.48	0.47
Diprotodontia	constant	g_{0R}	one profiled; glm fit rest of parameters	0.03	0.03	0.5	0.48	0.47

Eulipotyphla	constant	T_{wR}	one profiled; rest of parameters constant	1	1.36	0.66	0.9	0.54
Eulipotyphla	constant	T_{vS}	one profiled; rest of parameters constant	2	1.48	0.6	0.9	0.54
Eulipotyphla	constant	g_{0R}	one profiled; rest of parameters constant	0.04	0.06	0.55	0.9	0.54
Eulipotyphla	constant	T_{wR}	one profiled; glm fit rest of parameters	1	1.36	0.9	0.9	0.54
Eulipotyphla	constant	T_{vS}	one profiled; glm fit rest of parameters	2	1.48	0.8	0.9	0.54
Eulipotyphla	constant	g_{0R}	one profiled; glm fit rest of parameters	0.04	0.06	0.55	0.9	0.54
Perissodactyla	constant	T_{wR}	one profiled; rest of parameters constant	1	1.61	0.61	0.45	0.55
Perissodactyla	constant	T_{vS}	one profiled; rest of parameters constant	2	1.48	0.55	0.45	0.55
Perissodactyla	constant	g_{0R}	one profiled; rest of parameters constant	0.04	0.03	0.51	0.45	0.55
Perissodactyla	constant	T_{wR}	one profiled; glm fit rest of parameters	2	1.61	0.45	0.45	0.55
Perissodactyla	constant	T_{vS}	one profiled; glm fit rest of parameters	1	1.48	0.54	0.45	0.55
Perissodactyla	constant	g_{0R}	one profiled; glm fit rest of parameters	0.04	0.03	0.51	0.45	0.55
Primates	constant	T_{wR}	one profiled; rest of parameters constant	1	1.89	0.62	0.61	0.46
Primates	constant	T_{vS}	one profiled; rest of parameters constant	2	2	0.55	0.61	0.46
Primates	constant	g_{0R}	one profiled; rest of parameters constant	0.03	0.05	0.43	0.61	0.46
Primates	constant	T_{wR}	one profiled; glm fit rest of parameters	1	1.89	0.6	0.61	0.46
Primates	constant	T_{vS}	one profiled; glm fit rest of parameters	2	2	0.61	0.61	0.46
Primates	constant	g_{0R}	one profiled; glm fit rest of parameters	0.04	0.05	0.46	0.61	0.46
Rodentia	constant	T_{wR}	one profiled; rest of parameters constant	1	1.5	0.64	0.59	0.52
Rodentia	constant	T_{vS}	one profiled; rest of parameters constant	2	1.52	0.58	0.59	0.52
Rodentia	constant	g_{0R}	one profiled; rest of parameters constant	0.04	0.05	0.54	0.59	0.52
Rodentia	constant	T_{wR}	one profiled; glm fit rest of parameters	1	1.5	0.58	0.59	0.52
Rodentia	constant	T_{vS}	one profiled; glm fit rest of parameters	2	1.52	0.53	0.59	0.52
Rodentia	constant	g_{0R}	one profiled; glm fit rest of parameters	0.04	0.05	0.53	0.59	0.52
Carnivora	complete	T_{wR}	one profiled; rest of parameters constant	1	0.64	0.56	0.55	0.59
Carnivora	complete	T_{vS}	one profiled; rest of parameters constant	0.43	0.48	0.59	0.55	0.59
Carnivora	complete	g_{0R}	one profiled; rest of parameters constant	0.05	0.05	0.57	0.55	0.59

Carnivora	complete	T_{wR}	one profiled; glm fit rest of parameters	1	0.64	0.56	0.55	0.59
Carnivora	complete	T_{vS}	one profiled; glm fit rest of parameters	0.38	0.48	0.59	0.55	0.59
Carnivora	complete	g_{0R}	one profiled; glm fit rest of parameters	0.05	0.05	0.58	0.55	0.59
Cetartiodactyla	complete	T_{wR}	one profiled; rest of parameters constant	0	0.49	0.56	0.38	0.38
Cetartiodactyla	complete	T_{vS}	one profiled; rest of parameters constant	0.96	0.48	0.37	0.38	0.38
Cetartiodactyla	complete	g_{0R}	one profiled; rest of parameters constant	0.03	0.03	0.38	0.38	0.38
Cetartiodactyla	complete	T_{wR}	one profiled; glm fit rest of parameters	0.49	0.49	0.38	0.38	0.38
Cetartiodactyla	complete	T_{vS}	one profiled; glm fit rest of parameters	0.47	0.48	0.38	0.38	0.38
Cetartiodactyla	complete	g_{0R}	one profiled; glm fit rest of parameters	0.03	0.03	0.39	0.38	0.38
Chiroptera	complete	T_{wR}	one profiled; rest of parameters constant	1	1.01	0.57	0.92	0.92
Chiroptera	complete	T_{vS}	one profiled; rest of parameters constant	0	0.48	0.77	0.92	0.92
Chiroptera	complete	g_{0R}	one profiled; rest of parameters constant	0.07	0.07	0.85	0.92	0.92
Chiroptera	complete	T_{wR}	one profiled; glm fit rest of parameters	1	1.01	0.92	0.92	0.92
Chiroptera	complete	T_{vS}	one profiled; glm fit rest of parameters	0.47	0.48	0.92	0.92	0.92
Chiroptera	complete	g_{0R}	one profiled; glm fit rest of parameters	0.07	0.07	0.87	0.92	0.92
Diprotodontia	complete	T_{wR}	one profiled; rest of parameters constant	0	0.55	0.56	0.48	0.42
Diprotodontia	complete	T_{vS}	one profiled; rest of parameters constant	0.86	0.11	0.42	0.48	0.42
Diprotodontia	complete	g_{0R}	one profiled; rest of parameters constant	0.03	0.03	0.39	0.48	0.42
Diprotodontia	complete	T_{wR}	one profiled; glm fit rest of parameters	0	0.55	0.48	0.48	0.42
Diprotodontia	complete	T_{vS}	one profiled; glm fit rest of parameters	0.33	0.11	0.42	0.48	0.42
Diprotodontia	complete	g_{0R}	one profiled; glm fit rest of parameters	0.02	0.03	0.42	0.48	0.42
Eulipotyphla	complete	T_{wR}	one profiled; rest of parameters constant	0	0.36	0.59	0.82	0.49
Eulipotyphla	complete	T_{vS}	one profiled; rest of parameters constant	0.75	0.48	0.49	0.82	0.49
Eulipotyphla	complete	g_{0R}	one profiled; rest of parameters constant	0.04	0.06	0.49	0.82	0.49
Eulipotyphla	complete	T_{wR}	one profiled; glm fit rest of parameters	0	0.36	0.81	0.82	0.49
Eulipotyphla	complete	T_{vS}	one profiled; glm fit rest of parameters	1	0.48	0.52	0.82	0.49
Eulipotyphla	complete	g_{0R}	one profiled; glm fit rest of parameters	0.04	0.06	0.5	0.82	0.49

Perissodactyla	complete	T_{wR}	one profiled; rest of parameters constant	0	0.61	0.55	0.41	0.5
Perissodactyla	complete	T_{vS}	one profiled; rest of parameters constant	0.64	0.48	0.5	0.41	0.5
Perissodactyla	complete	g_{0R}	one profiled; rest of parameters constant	0.04	0.03	0.46	0.41	0.5
Perissodactyla	complete	T_{wR}	one profiled; glm fit rest of parameters	1	0.61	0.41	0.41	0.5
Perissodactyla	complete	T_{vS}	one profiled; glm fit rest of parameters	0.16	0.48	0.5	0.41	0.5
Perissodactyla	complete	g_{0R}	one profiled; glm fit rest of parameters	0.04	0.03	0.47	0.41	0.5
Primates	complete	T_{wR}	one profiled; rest of parameters constant	0	0.89	0.55	0.39	0.42
Primates	complete	T_{vS}	one profiled; rest of parameters constant	0.86	1	0.41	0.39	0.42
Primates	complete	g_{0R}	one profiled; rest of parameters constant	0.03	0.05	0.38	0.39	0.42
Primates	complete	T_{wR}	one profiled; glm fit rest of parameters	1	0.89	0.39	0.39	0.42
Primates	complete	T_{vS}	one profiled; glm fit rest of parameters	0.95	1	0.41	0.39	0.42
Primates	complete	g_{0R}	one profiled; glm fit rest of parameters	0.06	0.05	0.44	0.39	0.42
Rodentia	complete	T_{wR}	one profiled; rest of parameters constant	0	0.5	0.58	0.52	0.47
Rodentia	complete	T_{vS}	one profiled; rest of parameters constant	0.77	0.52	0.47	0.52	0.47
Rodentia	complete	g_{0R}	one profiled; rest of parameters constant	0.04	0.05	0.48	0.52	0.47
Rodentia	complete	T_{wR}	one profiled; glm fit rest of parameters	0	0.5	0.52	0.52	0.47
Rodentia	complete	T_{vS}	one profiled; glm fit rest of parameters	0.65	0.52	0.47	0.52	0.47
Rodentia	complete	g_{0R}	one profiled; glm fit rest of parameters	0.04	0.05	0.47	0.52	0.47

* Two sensitivity analyses were conducted: in the first, all within-host parameters were held constant across all mammalian orders at values listed in Table 1, excepting $\mu_R$which we varied by order according to values presented in S1 Table, and one additional parameter (either T_{wR}, g_{0R}, or T_{vS}) which we optimized. In these exercises, the parameter under optimization (listed here) was profiled until we recovered the best log-likelihood fit to the case fatality data recovered from the literature for that particular order. In the second exercise, parameter values for μ_R, T_{wR}, g_{0R}, and T_{vS} were varied across orders at values recovered from linear modeling of publicly available life history data, as presented in S1 Table. Here, we optimized one of three variable parameters (either T_{wR}, g_{0R}, or T_{vS}) further while holding all other parameters at S1 Table values. Resulting model fits and linear regression of observed vs. predicted values are visualized in S9 Fig.
