# Supplementary material for: Reservoir host immunology and life history shape virulence evolution in zoonotic viruses
Source: PLoS Biol. 2023 Sep 7;21(9):e3002268. doi: 10.1371/journal.pbio.3002268 (PMC10484437; doi:10.1371/journal.pbio.3002268)
Supplement: S1 File — (PDF) [file pbio.3002268.s013.pdf]

Supplementary Information Text

S1 Text. Population-level dynamics and R_0

To begin, we follow Miller et al. (2006) (1), assuming a simple population dynamics system which allows for infection-induced mortality. However, since we are interested in examining virus evolution in reservoir hosts, we first assume that hosts maintain persistent lifelong infections following initial exposure.

We assume that hosts are born at rate b_R and die of natural death at rate μ_R, where $b_R > \mu_R$. All hosts are assumed to be born susceptible and population density is regulated via a crowding term (q_R). The subscript "R" on the birth, death, and crowding terms emphasizes that these rates are specific to the reservoir host. Because our goal in this endeavor is to allow for the evolution of rates that link to within-host dynamics (Text S3), we represent transmission and virulence as functions of the virus causing the infection respectively, (β_{r_R} and α_{r_R}) where r_R denotes the intrinsic virus growth rate. Our system is described by the following system of ordinary differential equations (ODE), where 'S' represents hosts susceptible to infection and 'I' represents infected hosts and ($N = S + I$):

$$\frac{dS}{dt} = N(b_R - q_R N) - \beta_{r_R} SI - \mu_R S \quad [1a]$$

$$\frac{dI}{dt} = \beta_{r_R} SI - \mu_R I - \alpha_{r_R} I \quad [1b]$$

There exists both a disease-free equilibrium (DFE) at $(I^*_{DFE}, S^*_{DFE}) = (0, \frac{b_R - \mu_R}{q_R})$, as well as an endemic infection equilibrium (EE), (I^*_{EE}, S^*_{EE}) (see below, *[2a], [2b]*).

$$S^*_{EE} = \frac{\alpha_{r_R} + \mu_R}{\beta_{r_R}} \quad [2a]$$

$$I^*_{EE} = \frac{\sqrt{\beta_{r_R}\left[\beta_{r_R}\left(b_R - \mu_R - \alpha_{r_R}\right)^2 + 4\,\alpha_{r_R} q_R\left(\mu_R + \alpha_{r_R}\right)\right]} + \beta_{r_R}\left(b_R - \mu_R - \alpha_{r_R}\right) - 2q_R\left(\alpha_{r_R} + \mu_R\right)}{2\,\beta_{r_R} q_R} \quad [2b]$$

From *[1]*, we can then formulate the Jacobian matrix, J, to decipher the local stability of the DFE:

$$J = \begin{bmatrix} b_R - 2q_R S - 2q_R I - \beta_{r_R} I - \mu_R & b_R - 2q_R S - 2q_R I - \beta_{r_R} S \\ \beta_{r_R} I & \beta_{r_R} S - \mu_R - \alpha_{r_R} \end{bmatrix} \quad [3a]$$

Next, we evaluate the Jacobian at the DFE:

$$J_{DFE} = \begin{bmatrix} -b_R + \mu_R & -b_R + 2\mu_R - \frac{\beta_{r_R}(b_R - \mu_R)}{q_R} \\ 0 & \frac{\beta_{r_R}(b_R - \mu_R)}{q_R} - \mu_R - \alpha_{r_R} \end{bmatrix} \quad [3b]$$

The DFE is locally unstable when at least one of the eigenvalues of the Jacobian is non-negative. The eigenvalues can be read off the diagonal (*J_{DFE}* is upper triangular).

Clearly, the first eigenvalue is negative since $b_R > \mu_R$: $\mu_R - b_R < 0$. A little algebra will establish that the second eigenvalue is negative when:

$$R_0 = \frac{\beta_{r_R}(b_R - \mu_R)}{q_R(\mu_R + \alpha_{r_R})} < 1 \quad [4]$$

Therefore, if $R_0 > 1$, the DFE is no longer locally asymptotically stable, resulting in an increase in infected hosts within the population.

When $R_0 > 1$, the EE is globally asymptotically stable in the interior of $\Omega = \{(S, I) \in R_+^2 \mid S \geq 0, I \geq 0, S + I \leq \frac{b_R - \mu_R}{q_R}\}$. We demonstrate this by first establishing the local asymptotic stability of the EE, then by determining that there are no limit cycles in the region, Ω.

If we evaluate the Jacobian, once again, at the EE (which we can refer to now as J_{EE}), we will find that the trace *(Trace(J_{EE}))* of that matrix is negative:

$$Trace(J_{EE}) = \frac{-\left(2q_R + \beta_{r_R}\right)\sqrt{\beta_{r_R}\left[\beta_{r_R}\left(b_R - \mu_R - \alpha_{r_R}\right)^2 + 4\,\alpha_{r_R} q_R\left(\mu_R + \alpha_{r_R}\right)\right]} - \beta_{r_R}\left[\beta_{r_R}\left(b_R - \mu_R - \alpha_{r_R}\right) - 2q_R\left(\mu_R + \alpha_{r_R}\right)\right]}{2q_R\beta_{r_R}} < 0 \qquad [5]$$

Note that $b \geq \mu + \alpha_r$ (a necessary condition for the existence of the EE). In addition, the determinant of the J_{EE} is positive:

$$Det(J_{EE}) = \frac{\sqrt{\beta_{r_R}\left[\beta_{r_R}\left(b_R - \mu_R - \alpha_{r_R}\right)^2 + 4\,\alpha_{r_R} q_R\left(\mu_R + \alpha_{r_R}\right)\right]}\left[1 - 2q_R\left(\mu_R + \alpha_{r_R}\right)\right] + \beta_{r_R}\left(b_R - \mu_R - \alpha_{r_R}\right)}{2q_R\beta_{r_R}} > 0 \qquad [6]$$

Note that $0 < q_R \ll 1$. Therefore, the eigenvalues of the Jacobian matrix (J_{EE}) have negative real parts, and therefore the EE (when it exists) is locally asymptotically stable. We now use the Dulac criterion to establish that no limit cycles exist in Ω. Consider the Dulac function: $\Phi(S, I) = 1/(\beta S I)$, and let the function $F(S, I)$ represent the right hand side of *[1a]*, and let the function $G(S, I)$ represent the right hand side of *[1b]*. Then we have:

$$\frac{\partial(\Phi F)}{\partial S} + \frac{\partial(\Phi G)}{\partial I} = -\frac{(b_R)I}{\beta S^2 I} + q\,\frac{I^2 - S^2}{\beta S^2 I} < 0 \qquad [7]$$

since $0 < q_R \ll 1$. Thus, system *[1]* has no limit cycles present in Ω. Therefore, the EE is globally asymptotically stable in Ω when $R_0 > 1$.

S2 Text. Invasion of a mutant parasite under SI assumptions

Next, we aim to establish under what conditions a mutant pathogen can invade this system. We accomplish this task by first re-writing system *[1]* to include hosts infected with a resident wild-type pathogen, I_1, or a novel mutant pathogen, I_2 where now, $N = S + I_1 + I_2$:

$$\frac{dS}{dt} = N(b_R - q_R N) - \beta_{r_{1R}} S I_1 - \beta_{r_{2R}} S I_2 - \mu_R S \qquad [8a]$$
$$\frac{dI_1}{dt} = \beta_{r_{1R}} S I_1 - \mu_R I_1 - \alpha_{r_{1R}} I_1 \qquad [8b]$$
$$\frac{dI_2}{dt} = \beta_{r_{2R}} S I_2 - \mu_R I_2 - \alpha_{r_{2R}} I_2 \qquad [8c]$$

Notice that the novel mutant pathogen has a different intrinsic viral growth rate than that of the resident (wild-type) pathogen ($r_{1R} \neq r_{2R}$). It then follows from *[1]* that there exists a mutant-free equilibrium (MFE) in system *[8]* which recapitulates the endemic equilibrium in system *[1]*. To evaluate the local stability of the MFE we can again construct the Jacobian matrix of the new system and evaluate it at the MFE (Note: at the MFE, $I_2^* = 0$ while $S^*$and I_1^* replicate the endemic equilibrium values in *[2a]* and *[2b]*):

$$J_{MFE} = \begin{bmatrix} b_R - 2q_R S^* - 2q_R I_1^* - \beta_{r_{1R}} I_1^* - \mu_R & b_R - 2q_R S^* - 2q_R I_1^* - \beta_{r_{1R}} S^* & b_R - 2q_R S^* - 2q_R I_1^* - \beta_{r_{2R}} S^* \\ \beta_{r_{1R}} I_1^* & \beta_{r_{1R}} S^* - \mu_R - \alpha_{r_{1R}} & 0 \\ 0 & 0 & \beta_{r_{2R}} S^* - \mu_R - \alpha_{r_{2R}} \end{bmatrix} \quad [9]$$

The upper left four quadrats of this matrix exactly replicate the 2x2 Jacobian (*[3a]*) evaluated at the EE (J_{EE}), which we now refer to as the resident equilibrium. J_{MFE} is a block-triangular matrix, which we can be broken down into component parts:

$$J_{MFE} = \begin{bmatrix} J_{res} & * \\ 0 & J_{mut} \end{bmatrix} \quad [10]$$

Stability of the MFE requires that the real parts of all eigenvalues of J_{MFE} be negative. Since the matrix is block diagonal, the eigenvalues of J_{MFE} are given by the eigenvalues of the two matrices on the diagonal, J_{res} and J_{mut}. We already established that the eigenvalues of J_{res} have negative real parts when $R_0 > 1$. The local stability of the MFE then depends on the sign of the eigenvalue of J_{mut}, where $J_{mut} = \beta_2 S_{EE}^* - \mu_R - \alpha_{r_{2R}}$. J_{mut} thus corresponds to the growth rate of a rare mutant in an environment with an endemic resident strain. If the mutant pathogen is able to grow within the population, then:

$$\beta_{r_{2R}} \frac{\mu_R + \alpha_{r_{1R}}}{\beta_{r_{1R}}} - \mu_R - \alpha_{r_{2R}} > 0 \quad [11]$$

or:

$$\frac{\beta_{r_{2R}}}{\mu_R + \alpha_{r_{2R}}} > \frac{\beta_{r_{1R}}}{\mu_R + \alpha_{r_{1R}}} \quad [12]$$

It is evident that the MFE is unstable (allowing for invasion of the mutant strain) when the ratio of transmission over infection duration is larger for the (invading) mutant strain than the resident strain. In general, a pathogen will evolve to maximize this expression, which is known as the invasion fitness, and is given by:

$$\frac{\beta_{r_R}}{\mu_R + \alpha_{r_R}} \quad [13]$$

S3 Text. Within-host dynamics under SI assumptions

Now that we have determined the invasion fitness for a mutant virus, we can write the population-level rates dependent on within-host dynamics in their within-host terms (transmission: $\beta_{r_{1R}/r_{2R}}$ and virulence: $\alpha_{r_{1R}/r_{2R}}$). This will allow us to derive a selection gradient on the viral growth rate, an intrinsic virus property likely to be carried from one host to another. To do this, we establish a simple within-host model that represents the dynamics of infection *within* each I_1 and I_2 host as outlined above. We follow Alizon and Van Baalen (2005) (2) to adapt a class of Lotka-Volterra predator-prey-like within-host models (reviewed in (3)), which overcomes some of the constraints of basic predator-prey models of the immune system, chiefly *(i)* by allowing leukocytes to circulate in the absence of infection and *(ii)* by scaling leukocyte growth with virion density, independent of direct leukocyte-virion contact (2,4,5) . This model can be captured by the following system of ODEs, which demonstrates interactions between the virus population (V_R) and the leukocyte population (L_R) within each infected reservoir host:

$$\frac{dV_R}{dt} = r_R V_R - c_R V_R L_R \quad [14a]$$
$$\frac{dL_R}{dt} = g_{0R} + g_R r_R V_R - m_R L_R \quad [14b]$$

where r_R corresponds to the intrinsic virus growth rate, c_R corresponds to the attack efficacy of the immune system upon contact with the virus, and g_R signifies the recruitment rate of immune cells scaled

to the virus growth rate. The parameter, g_{0R}, describes the constitutive, baseline leukocyte recruitment in the absence of infection, and m_R gives the natural leukocyte death rate.

This system has two equilibria: a virus free equilibrium at $V_R^* = 0$ and $L_R^* = \frac{g_{0R}}{m_R}$ and an endemic virus equilibrium at $V_R^* = \frac{m_R r_R - c_R g_{0R}}{g_R c_R r_R}$ and $L_R^* = \frac{r_R}{c_R}$.

Building from above, we can then rewrite the component terms of the population-level transmission and virulence rates dependent on within-host dynamics ($\beta_{r_{1R}/r_{2R}}$ and $\alpha_{r_{1R}/r_{2R}}$) in terms of their within-host components, assuming that the within host dynamics are fast relative to the population-level dynamics and converge to the endemic equilibrium (i.e. $m_R r_R > c_R g_{0R}$).

In line with previous work, we assume transmission to be a linear function of viral load (4), which we represent as:

$$\beta_{r_R} = \zeta V_R^*. \quad [15]$$

We can also assume that:

$$\alpha_{r_R} = \frac{v r_R V_R^*}{T_{vR}} + \frac{w g_R r_R V_R^*}{T_{wR}}, \quad [16]$$

by which infection-induced host mortality ('virulence') results from both virus-induced pathology, a function of the intrinsic virulence of the parasite, v, and the parasite growth rate $r_R V_R^*$, and immunopathology, which we model as proportional to the leukocyte growth rate in response to virus expansion, multiplied by the parasite's intrinsic propensity to elicit an inflammatory immune response, given here as w. The terms T_{vR} and T_{wR} correspond to host tolerance of virus-induced pathology and immunopathology, respectively.

Expression *[16]* assumes a form of "constant" tolerance, by which both virus pathology and immunopathology are reduced by a constant proportion across the course of infection. For constant tolerance, we assume that $T_{vR} > 1$ and $T_{wR} > 1$. After Miller et al. 2006 (1), we can also model these relationships assuming "complete tolerance" *[17]*, whereby virus pathology and immunopathology are completely eliminated up to a threshold value, beyond which pathology scales proportionally with virus and immune cell growth:

$$\alpha_{r_R} = (v - T_{vR}) r_R V_R^* + (w - T_{wR}) g_R r_R V_R^* \quad [17]$$

For complete tolerance, we assume that $0 < T_{vR} < v$ and $0 < T_{wR} < w$.
We consider both constant and complete forms of tolerance in all subsequent analyses. It is important to note that the predictions of the complete form of tolerance are not dependent on the assumption of complete elimination: a more gradual function shows equivalent results (1).

Now, we can rewrite the above equations for transmission (*[15]*) and virulence (*[16]*/*[17]*) in purely within-host terms, here for constant tolerance:

$$\beta_{r_R} = \frac{\zeta(m_R r_R - c_R g_{0R})}{c_R g_R r_R} \quad [18]$$

$$\alpha_{r_R} = \frac{v(m_R r_R - c_R g_{0R})}{c_R g_R T_{vR}} + \frac{w(m_R r_R - c_R g_{0R})}{c_R T_{wR}} \quad [19]$$

And here for complete tolerance (note that only the equation for virulence—but not transmission—differs under differing forms of tolerance):

$$\alpha_{r_R} = \frac{(m_R r_R - c_R g_{0R})(v - T_{vR})}{c_R g_R} + \frac{(m_R r_R - c_R g_{0R})(w - T_{wR})}{c_R} \quad [20]$$

We can then allow the virus to evolve its within-host growth rate (r_R) to maximize its reproductive success (*[13]* for the SI model above). In within-host terms, the invasion fitness takes on the following general form, under assumptions of constant tolerance:

$$\frac{\zeta T_{vR} T_{wR}(m_R r_R - c_R g_{0R})}{r_R T_{wR}(c_R g_R \mu_R T_{vR} - c_R g_{0R} v + m_R r_R v) + g_R r_R w T_{vR}(m_R r_R - c_R g_{0R})} \qquad [21]$$

And under assumptions of complete tolerance:

$$\frac{\zeta(c_R g_{0R} - m_R r_R)}{r_R\left[m_R r_R(T_{vR} - v + g_R T_{wR} - g_R w) - c_R\left(g_{0R}(v - T_{vR}) + g_R(\mu_R + g_{0R} T_{wR} - g_{0R} w)\right)\right]} \qquad [22]$$

We can then determine the selection gradient on r by differentiating *[21]* and *[22]* with respect to r_R. This gives us the following expression under assumptions of constant tolerance (differentiating *[21]*):

$$-\frac{\zeta T_{vR} T_{wR}[g_R w T_{vR}(c_R g_{0R} - m_R r_R)^2 + v T_{wR}(c_R g_{0R} - m_R r_R)^2 - c_R^2 g_R g_{0R}^2 \mu_R T_{vR} T_{wR}]}{[r_R T_{wR}(c_R g_R \mu_R T_{vR} - c_R g_{0R} v + m_R r_R v) + g_R r_R w T_{vR}(m_R r_R - c_R g_{0R})]^2} \qquad [23]$$

And assuming complete tolerance (differentiating *[22]*):

$$\frac{\zeta\left[m_R^2 r_R^2(T_{vR} - v + g_R T_{wR} - g_R w) + 2 c_R g_{0R} m_R r_R(v - T_{vR} + g_R w - g_R T_{wR}) + c_R^2 g_{0R}\left(g_{0R}(T_{vR} - v) + g_R(\mu + g_{0R} T_{wR} - g_{0R} w)\right)\right]}{r_R^2\left[c_R g_{0R}(T_{vR} - v) + c_R g_R\left(\mu_R + g_{0R}(w - T_{wR})\right) + m_R r_R\left(v - T_{vR} + g_R(w - T_{wR})\right)\right]^2} \qquad [24]$$

We then set these derivatives equal to 0 and solve for *r*, which we now refer to as r^*. The parameter r^* corresponds to the optimal within-host virus growth rate, here under assumptions of constant tolerance:

$$r_R^* = \frac{c_R g_{0R}}{m_R} + \frac{\sqrt{c_R^2 m_R^2 g_R g_{0R} \mu_R T_{vR} T_{wR}(v T_{wR} + g_R w T_{vR})}}{m_R^2(v T_{wR} + g_R w T_{vR})} \qquad [25]$$

And here under assumptions of complete tolerance:

$$r_R^* = \frac{c_R g_{0R}}{m_R} + \frac{c_R^2 g_R g_{0R} \mu_R}{\sqrt{c_R^2 m_R^2 g_R g_{0R} \mu_R(v - T_{vR} + g_R w - g_R T_{wR})}} \qquad [26]$$

We can check that this optimum is a locally stable maximum by proving that the second derivative of the invasion fitness (*[21]* and *[22]*) with respect to r_R is negative at the respective values for r_R^* derived in *[25]* and *[26]*. When we do this, we find that this is, indeed, true, under assumptions of both constant and complete tolerance. This means that *[25]* and *[26]* represent true evolutionarily stable strategies (ESS). Furthermore, we can compute a pairwise invasibility plot to visually demonstrate that the derived expression for r_R^* represents an *ESS* (S1 Fig).

In S1 Fig, we see that all points along the vertical line through the point of intersection fall within the unshaded regions, corresponding to the conditions for a locally stable ESS (6). Additionally, we see that higher degrees of tolerance shift r_R^* higher under both constant and complete tolerance assumptions.

Finally, we can express *[16]* and *[17]* in within-host terms to calculate the virulence which a virus incurs on the reservoir host when evolved to its optimal within-host virus growth rate (r_R^*). When we do this, we find that virus-induced virulence at r_R^* (which we call $\alpha^*_{r_R}$) can be expressed as the following, under assumptions of constant tolerance:

$$\alpha^*_{r_R} = \frac{c_R g_{0R} m_R \mu_R(v T_{wR} + g_R w T_{vR})}{\sqrt{c_R^2 m_R^2 g_R g_{0R} \mu_R T_{vR} T_{wR}(v T_{wR} + g_R w T_{vR})}} \qquad [27]$$

And the following, under assumptions of complete tolerance:

$$\alpha^*_{r_R} = \frac{\sqrt{c_R^2 m_R^2 g_R g_{0R} \mu_R (v - T_{vR} + g_R w - g_R T_{wR})}}{c_R g_R m_R} \qquad [28]$$

There are several important insights to come out of these simple expressions for optimal viral growth r^*_R and corresponding virulence $\alpha^*_{r_R}$ in a reservoir host. We highlight the relationships between r^*_R and $\alpha^*_{r_R}$ and specific within-host parameters under assumptions of constant tolerance in Fig 2 (main text) and under assumptions of complete tolerance in S2 Fig.

S4 Text. Spillover virulence in the secondary host

Our primary interest in understanding the evolution of the optimal virus growth rate in a reservoir host is to predict the virulence that such a virus is likely to cause upon spillover to a novel, particularly human, host. Contrary to its established persistent infection in the reservoir host, we assume that the virus will produce an acute infection in the spillover host. To model the dynamics of this acute spillover, we can borrow from Gilchrist and Sasaki 2002 (7), who developed a within-host parasite-leukocyte model in which an immortal leukocyte successfully eradicates the parasite population to near-zero. Here, we modify their acute model to make more comparable to our chronic infection model (*[14a]/[14b]*) and to reflect our differing notation for within-host dynamics.

$$\frac{dV_S}{d\tau} = r^*_R V_S - c_S V_S L_S \qquad [29a]$$

$$\frac{dL_S}{d\tau} = r^*_R g_S V_S \qquad [29b]$$

where r^*_R represents the reservoir-evolved virus growth rate, but all other terms reflect the within-host conditions of the spillover host: V_S and L_S correspond, respectively, to the spillover host virus and leukocyte populations, c_S is the virus consumption rate upon contact with leukocytes in the spillover host, and g_S is the growth rate of the spillover host leukocyte population in response to virus. Here, the model is expressed in units of τ, which we assume to be short in comparison to the t time units of dynamics at play in the reservoir host population. Rather than solving for virus and leukocyte populations at equilibrium (as in the reservoir host population), we followed Gilchrist and Sasaki 2002 (7) to instead seek to derive an expression for the virus population at the peak of infection ($V_{S_{max}}$), where we anticipate maximum pathology for the spillover host. We then extend this prior work to generate an expression for the average viral load ($V_{S_{avg}}$) in the spillover host, which we use in comparisons of spillover host virulence with reported case fatality rates for zoonoses in the literature.

If we divide equation *[29a]* by equation *[29b]* we can derive a simple time-independent relationship between virus and leukocyte density:

$$\frac{dV_S}{dL_S} = \frac{1}{g_S}\left(1 - \frac{c_S L_S}{r^*_R}\right) \qquad [30]$$

If we then let $L_S(0) = 1, V_S(0) = 1$, assuming that both virus and leukocyte populations will be small at $\tau = 0$, we can integrate *[30]* to establish the following relationship between virus and leukocyte:

$$V_S = \frac{1}{g_S} L_S - \frac{c_S}{2 r^*_R g_S}(L_S)^2 + 1 - \frac{1}{g_S} + \frac{c_S}{2 r^*_S g_S} \qquad [31]$$

Notice that the above *[31]* is simply a quadratic equation (see *[32]* below):

$$V_S = -\frac{c_S}{2 r^*_R g_S}(L_S)^2 + \frac{1}{g_S} L_S + \left(1 - \frac{1}{g_S} + \frac{c_S}{2 r^*_S g_S}\right) \qquad [32]$$

Now, we can take the derivative of equation *[32]* to formulate an expression for $V_{S_{max}}$, the maximum viral load, which should precede the end of acute infection and the point of host recovery at the maximum duration of infection:

$$V_{S_{max}} = \frac{r_R^*}{g_S c_S} - \frac{r_R^*}{2 g_S c_S} + 1 - \frac{1}{g_S} + \frac{c_S}{2 r_R^* g_S} \qquad [33]$$

Now, extending previous work (7), we can calculate the average viral load by taking the integral of equation *[32]* from $V_S(0)$ to $V_{S_{max}}$ and dividing by the duration of that interval. From this exercise, we can express the average value of equation *[32]* as:

$$V_{S_{avg}} = \frac{r_R^* \sqrt{c_S^2 + 2 r_R^* (g_S - 1) c + \left(r_R^*\right)^2} + (r_R^*)^2 + 4 r_R^* (g-1) c + 2 c_S^2}{6 c_S g_S r_R^*} \qquad [34]$$

Now, with this established expression for $V_{S_{avg}}$, we can adapt equations *[16]* and *[17]* to reflect the acute within-host dynamics of “spillover virulence”, which we model (as before) as a combination of mortality induced from direct virus pathology and from immunopathology. As in the reservoir host system, we can also model the mitigating impact of tolerance on the two mechanisms of virulence, here under assumptions of constant tolerance:

$$\alpha_S = V_{S_{avg}} \left(\frac{r_R^* v}{T_{vS}} + \frac{g_S w r_R^*}{T_{wS}} \right) \qquad [35]$$

In our expression for spillover virulence, we model the growth rate of the virus at its evolutionary optimum evolved in the reservoir host (r_R^*), and we represent a virus’s intrinsic virulence (v) and propensity to elicit an inflammatory immune response (w) as unchanged from one host to the next. Inspired by findings in the literature that report higher virulence in cross-species infections between hosts separated by larger phylogenetic distances (8–12), we model spillover host tolerance of virus-induced pathology (T_{vS}) as a decreasing function of increasing phylogenetic distance between the reservoir and secondary host. All other immune-related parameters take on characteristics of the spillover host: g_S is the spillover host’s leukocyte growth rate, and T_{wS} corresponds to the spillover host’s tolerance of immunopathology.

We can also represent spillover virulence under assumptions of complete tolerance:

$$\alpha_S = V_{S_{avg}} \left(r_R^* (v - T_{vS}) + r_R^* g_{SR} (w - T_{wS}) \right) \qquad [36]$$

SI References

1. Miller MR, White A, Boots M. The evolution of parasites in response to tolerance in their hosts: The good, the bad, and apparent commensalism. Evolution. 2006;60(5):945.

2. Alizon S, van Baalen M. Emergence of a convex trade-off between transmission and virulence. The American Naturalist. 2005;165(1537-5323 (Electronic)):E155–67.

3. Alizon S, van Baalen M. Acute or Chronic? Within-host models with immune dynamics, infection outcome, and parasite evolution. The American Naturalist. 2008;172(6):E244–56.

4. Alizon S, van Baalen M. Multiple infections, immune dynamics, and the evolution of virulence. The American Naturalist. 2008;172(4):E150–68.

5. Fenton A, Lello J, Bonsall MB. Pathogen responses to host immunity: The impact of time delays and memory on the evolution of virulence. Proceedings of the Royal Society B: Biological Sciences. 2006;273(1597):2083–90.

6. Geritz SAH, Kisdi É, Meszéna G, Metz JAJ. Evolutionarily singular strategies and the adaptive growth and branching of the evolutionary tree. Evolutionary Ecology. 1998;12(1):35–57.

7. Gilchrist MA, Sasaki A. Modeling host – parasite coevolution: A nested approach based on mechanistic models. Journal of Theoretical Biology. 2002;218:289–308.
